# Structural and Morphological Tuning of LiCoPO_4_ Materials Synthesized by Solvo-Thermal Methods for Li-Cell Applications

**DOI:** 10.3390/nano5042212

**Published:** 2015-12-10

**Authors:** Jessica Manzi, Mariangela Curcio, Sergio Brutti

**Affiliations:** 1 Department of Science, University of Basilicata, V.le Ateneo Lucano 10, Potenza 85100, Italy; E-Mails: jessica.manzi@unibas.it (J.M.); mariangela.curcio@unibas.it (M.C.); 2 CNR-ISC, U. O. S. La Sapienza, Piazzale A. Moro 5, 00185 Roma, Italy

**Keywords:** LiCoPO_4_, olivine, cathode materials, Li batteries, solvo-thermal synthesis

## Abstract

Olivine-type lithium metal phosphates (LiMPO_4_) are promising cathode materials for lithium-ion batteries. LiFePO_4_ (LFP) is commonly used in commercial Li-ion cells but the Fe^3+^/Fe^2+^ couple can be usefully substituted with Mn^3+^/Mn^2+^, Co^3+^/Co^2+^, or Ni^3+^/Ni^2+^, in order to obtain higher redox potentials. In this communication we report a systematic analysis of the synthesis condition of LiCoPO_4_ (LCP) using a solvo-thermal route at low temperature, the latter being a valuable candidate to overcome the theoretical performances of LFP. In fact, LCP shows higher working potential (4.8 V *vs.* 3.6 V) compared to LFP and similar theoretical capacity (167 mAh·g^−1^). Our goal is to show the effect of the synthesis condition of the ability of LCP to reversibly cycle lithium in electrochemical cells. LCP samples have been prepared through a solvo-thermal method in aqueous-non aqueous solvent blends. Different Co^2+^ salts have been used to study the effect of the anion on the crystal growth as well as the effect of solution acidity, temperature and reaction time. Materials properties have been characterized by Fast-Fourier transform infrared spectroscopy, X-ray diffraction and scanning electron microscopies. The correlation between structure/morphology and electrochemical performances has been investigated by galvanostatic charge-discharge cycles.

## 1. Introduction

In the last decades, rechargeable lithium-ion batteries have evolved in terms of energy density, cycle life, and reliability [1] to match the increasing requirements of the commercial applications (*i.e.*, portable electronics, power tools). Further advance is needed in order to go beyond the typical application markets of Li-ion cells. A successful massive implementation of these energy storage devices in electric vehicles requires a huge jump in the performance, safety, and calendar life [2]. One way to increase battery energy is the use of high-voltage cathode materials [3].

Olivine compounds (LiMPO_4_) are interesting cathode materials for lithium-ion batteries: LiFePO_4_ (LFP) is commonly used in commercial Li-ion cells [4]. However, in the olivine structure the Fe^3+^/Fe^2+^ couple can be usefully substituted with the Mn^3+^/Mn^2+^, Co^3+^/Co^2+^, or Ni^3+^/Ni^2+^ ones in order to obtain higher redox potentials [5]. In particular, LiCoPO_4_ (LCP) is a valuable candidate to overcome the theoretical performances of LFP. It shows higher working potential (4.8 V *vs.* 3.6 V) compared to LFP and similar theoretical capacity (167 mAh·g^−1^) [5].

In a previous publication we reported an innovative synthetic route based on a solvo-thermal method to precipitate sub-micrometric platelet-like particles of LCP with excellent phase and morphological purities [6]. Starting from this synthesis we have also shown the beneficial partial substitution of cobalt with iron [7], studied its self-discharge [8], and investigated the incorporation of this enhanced material in full lithium-ion configuration with an ionic-liquid based electrolyte [9].

In this communication we report a more detailed and careful analysis of the synthetic conditions for the precipitation of LCP materials at low temperature (220 °C) by a solvo-thermal route. Our scope is the development of a synthetic strategy capable to tune the particle morphology and the structural properties of the LCP material and check the effect of the different synthetic condition on the ability to reversibly cycle lithium in electrochemical cells. For this purpose, LCP has been prepared by adapting the strategy developed in our laboratory [6]. In particular, here we illustrate the effect of the use of different Co^2+^ sources, solution acidity, and reaction time on the crystal growth of the LCP particles by means of a multi-technique approach and we report the corresponding performances in lithium cells.

## 2. Results and Discussion

### 2.1. Synthesis of the LCP Materials

**Figure 1:**
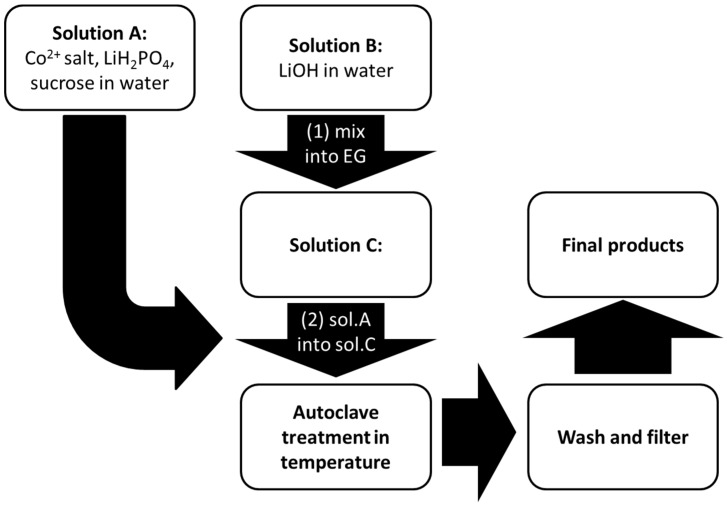
Schematic diagram of the synthesis route of LiCoPO_4_ (LCP).

LCP materials have been synthesized by following a solvo-thermal route [6]. A flowchart of the synthetic procedure is shown in the Figure 1.

In the first step, two aqueous solutions containing LiH_2_PO_4_, sucrose (as a reducing agent to prevent possible oxidation of Co^2+^ to Co^3+^), and a Co^2+^ salt precursor (solution A), and LiOH (solution B) respectively, have been prepared under vigorous stirring. The amount of sucrose has always been set in respect to the cobalt precursor with a molar ratio Co^2+^:sucrose = 1:0.03. The relative amount of LiOH has been optimized in order to obtain a moderately-acidic solution to facilitate the precipitation of the olivine-phosphate. More details are discussed in the Results section. Subsequently the solution B has been dissolved in ethylene glycol (EG) to give solution C. The total H_2_O:EG volume ratio has been set to 1:2. Afterward, solution A has been added dropwise into solution C under stirring. The final slurry (solution D) has been poured into a Teflon vessel in a stainless steel autoclave. The final volume of the final solution has been set to 30 mL with a nominal [Co^2+^] concentration of 0.1 M. After sealing, the autoclave has been kept in an oven at 200–240 °C for few hours and then allowed to cool naturally to room temperature.

The resulting suspension has been filtered and the solid pinkish precipitate has been washed in deionized water and ethanol, and then oven-dried at 80 °C. Three different reaction temperatures (200 °C, 220 °C, and 240 °C) have been investigated and the reaction time optimized to each temperature condition (see next section). Four different Co^2+^ sources have been used: Co(NO_3_)_2_, CoSO_4_, Co(CH_3_COO)_2_, and CoCO_3_. A variant of this synthesis route has been developed in the case of LCP from carbonate. Due to the low K_sp_ (1 × 10^−10^ at 25 °C) of the cobalt (II) carbonate, the Co^2+^ precursor has been directly suspended in EG. Therefore, starting solutions are A (CoCO_3_ in EG), B (sucrose and LiH_2_PO_4_ in H_2_O), and C (LiOH in H_2_O). Solution B has been dropped into solution D, obtained by joining A and C.

### 2.2. Analysis of the Synthesis Conditions: Effect of the Solution Acidity

**Figure 2:**
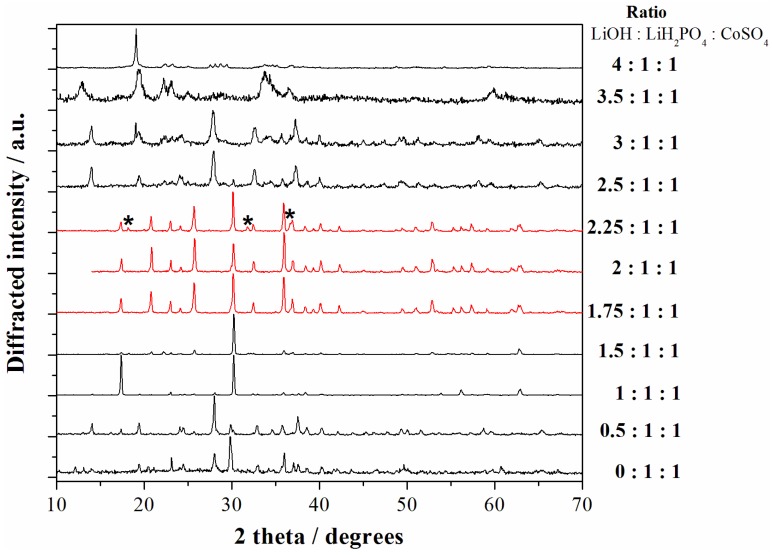
X-ray diffraction (XRD) patterns of all the samples obtained starting from the reagent ratios LiOH:LiH_2_PO_4_:CoSO_4_ ranging from 0:1:1 to 4:1:1. The red patterns correspond to the LCP reference diffractograms ((*) indicate peaks from impurities) whereas black patterns correspond to other reaction products. All materials have been obtained at 240 °C with solvo-thermal treatments of 5 h.

As mentioned above, the nature and ratio of the reagents, and in particular the amount of LiOH in the starting mixture, have been set in order to obtain a slightly acidic solution to facilitate the precipitation of the olivine phosphate. The precipitation of pure LCP samples from solutions at low temperature is a challenging task [10]. Delacourt *et al.* in reference [10] calculated the solid-state repartition diagram in water as a function of pH and cobalt(II) concentration, thus highlighting an optimal pH region in aqueous solution for the precipitation of LCP (pH range 6.3–9.5). However their attempt to precipitate the pure LCP phase from water solutions failed due to the apparently unavoidable precipitation of competing phases, such as Co_3_(PO_4_)_2_, Li_3_PO_4_, and Co(OH)_2_ [10]. Our case is even more complex due to the use of a mixed EG-H_2_O solvent blend. In this view we performed an experimental screening of the [LiOH]/[LiH_2_PO_4_]/[Co^2+^] ratio in the case of the cobalt sulfate salt in order to identify the most suitable range to obtain phase pure LCP particles. The X-ray diffraction (XRD) patterns of all the samples obtained starting from the reagent ratios LiOH:LiH_2_PO_4_:CoSO_4_ ranging from 0:1:1 to 4:1:1 are shown in the Figure 2.

Apparently the precipitation of the LCP phase (pure or almost pure) can be obtained in the LiOH:LiH_2_PO_4_:CoSO_4_ ratios range within 1.75:1:1 and 2.25:1:1. Our results are therefore in agreement with the evaluation for water solution from Delancourt *et al.* [10]. In particular, neutral (reagents ratios 2:1:1) and moderately acidic pH (reagents ratio 1.75:1:1) lead to phase pure samples constituted by LCP without impurities. In fact the lack of unindexed peaks in the corresponding XRD patterns confirms the phase purity of these two samples and the absence of contaminant phases above 2%–3% in volume, which is the typically accepted detection limit for phase identification by powder XRD [11]. All the other precipitation conditions lead to formation of other reaction products and have been, therefore, discarded.

On the other hand, from the point of view of the final elemental composition the two LCP-phase pure samples obtained in moderately acidic or neutral pH are not equivalent. In fact, whereas the material prepared starting from a reagent ratio LiOH:LiH_2_PO_4_:CoSO_4_ = 2:1:1 shows an experimental Li:Co ICP-AES ratio of 1.06:0.98, the material prepared in moderately acidic conditions (LiOH:LiH_2_PO_4_:CoSO_4_ = 1.75:1:1) has an almost stoichiometric LiCoPO_4_ experimental composition (Li:Co = 1.01:1.00). The difference in the elemental ratios between the samples prepared in neutral or moderately acidic conditions may imply the precipitation of minor contaminant phases in the first case, below the abovementioned detection limit of the XRD technique, or off-stoichiometric defects in the olivine lattice (see next section).

### 2.3. Analysis of the Synthesis Conditions: Effect of the Reaction Time

**Figure 3:**
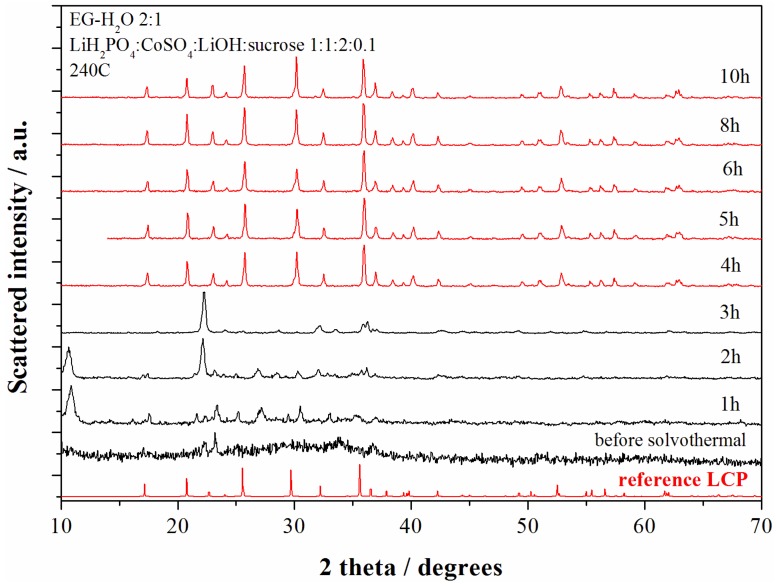
XRD patterns of the samples obtained starting from the reagent ratio LiOH:LiH_2_PO_4_:CoSO_4_ = 2:1:1 at 240 °C for different reaction time. The red patterns correspond to the LCP reference diffractograms whereas black patterns correspond to other reaction products.

The evolution of the phase composition of the precipitates obtained from the solvo-thermal bath at 240 °C have been studied upon time for the reagent ratios LiOH:LiH_2_PO_4_:CoSO_4_ = 2:1:1 (neutral pH conditions). The XRD patterns of the materials obtained at various reaction time are shown in the Figure 3.

The pristine product (before solvo-thermal treatment) obtained at the end of the starting solution mixing (violet precipitate) reveals an amorphous product with few broad unindexed peaks. After 1 h of solvo-thermal treatment Bragg reflections from crystalline phases start to become. After 4 h of reaction time at 240 °C the XRD pattern shows only reflections from the olivine LCP and no further structural evolution is observed up to 10 h of reaction.

The composition of the synthesized materials show a constant evolution with the reaction time. In fact the Li:Co ratio decreases monotonically from 1.85 (1 h), 1.37 (2 h), 1.15 (3 h), 1.10 (4 h), 1.08 (5 h), to 1.04 (10 h). The lithium excess in the LCP-pure samples (4–5–6–8–10 h) may suggest the presence of minor contaminants (below the XRD detection limit) or over-lithiation of the LCP olivine lattice.

**Figure 4:**
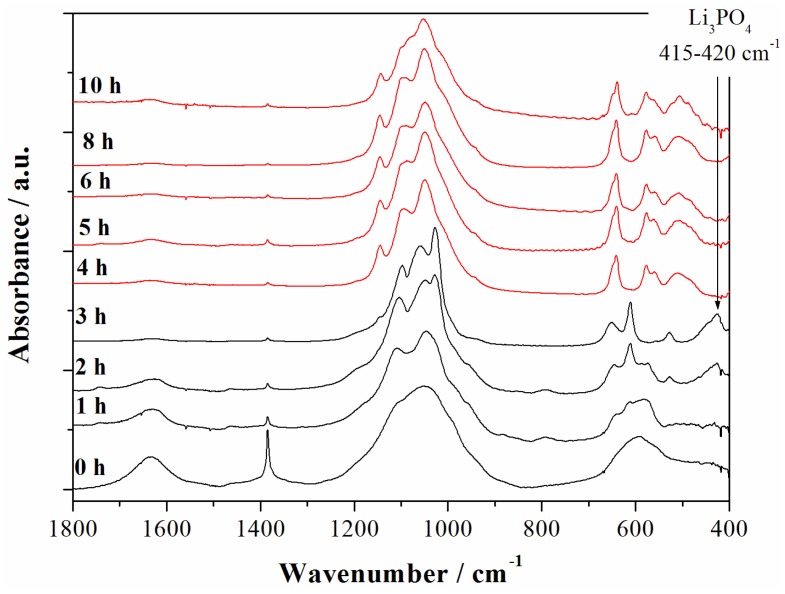
Fast-Fourier Transform infrared spectroscopy (FTIR) spectra of the samples obtained starting from the reagent ratios LiOH:LiH_2_PO_4_:CoSO_4_ = 2:1:1 at 240 °C for different reaction time. The red spectra correspond to the LCP reference one from reference [6] whereas black patterns correspond to other reaction intermediates.

In order to check the possible precipitation of minor contaminant species in the synthesized materials the evolution of the Fast-Fourier Transform infrared spectroscopy (FTIR) spectra of the materials prepared at various reaction time have been studied as shown in the Figure 4.

The strong water O–H bending mode at 1630–1640 cm^−1^ is observed in the early synthesis stages (0–2 h) whereas it disappears almost completely after the formation of the LCP phase. The spectrum of the material after 4 h of reaction time matches perfectly the reference one for the phase pure LCP olivine lattice [6] and remains almost unaltered for longer reaction time. In particular the intramolecular stretching modes of the PO_4_^3−^ anions are observed at (*ν*_3_) 1180, 1100, 1050, and (*ν*_1_) 975 cm^−1^, whereas the bending bands (*ν*_2_, *ν*_4_, and *ν*_2_ + *ν*_4_) are found at 640, 575, 550, 505, and 475 cm^−1^. Additional bands are not observed in all LCP-pure samples (4, 5, 6, and 8–10 h). In particular, at 410–420 cm^−1^ the typical fingerprint of the Li_3_PO_4_ phase is missing [7], as well as absorption bands in the 650 cm^−1^ to 940 cm^−1^ region, where vibrations associated to other phosphate anions, such as P_2_O_7_^4−^ [7], are located. In this view, considering the above reported results from the ICP-AES analysis for the trend of the Li:Co ratio upon reaction time, the absence of evident contaminations may be an indirect clue of the possible over-lithiation of the olivine lattice of samples prepared at 240 °C in neutral pH conditions. The excess of lithium incorporated in the material apparently decreases with the increase of the solvo-thermal reaction time.

Having established the absence of contaminant phases for all samples prepared at 240 °C after 4 h of reaction time and having measured the Li:Co ratios, it is possible to estimate a tentative stoichiometry for the corresponding LCP phase with the assumption of the electro-neutrality constraints between cations (Li^+^, Co^2+^) and anions (PO_4_^3−^) in the lattice. The resulting estimated stoichiometries are summarized in the Table 1 together with the results of the Rietveld refinement fitting performances on the corresponding XRD patterns by the GSAS code [12].

Cell constants are in agreement with literature values [5]: lattice parameters are slightly expanded compared to both annealed samples (approximately +0.6%) and to single crystals (approximately +1.2%) [5]. However, a clear structural evolution trend with the reaction time is missing, besides a slight increase of the overall cell volume for samples prepared for 8 h and 10 h. Similarly the structural disorder, *i.e.*, Li/Co anti-site defects [4], occurs in all samples without a monotonic trend.

**Table 1:** Estimated stoichiometries and Rietveld refinement results for the samples prepared at 240 °C in neutral pH conditions at various reaction time.

Reaction Time	Estimated Stoichiometry	a/Å	b/Å	c/Å	V/Å^3^ ± 0.2	%_Me Disorder_ ± 0.6	_w_*R*_p_
4 h	Li_1.06_Co_0.97_PO_4_	10.211(1)	5.924(2)	4.705(3)	284.6	3.1	7.3%
5 h	Li_1.05_Co_0.97_PO_4_	10.208(2)	5.923(2)	4.704(3)	284.4	2.7	8.0%
6 h	Li_1.05_Co_0.98_PO_4_	10.210(3)	5.924(2)	4.705(4)	284.6	2.6	6.5%
8 h	Li_1.03_Co_0.98_PO_4_	10.214(2)	5.928(1)	4.705(2)	284.9	2.8	6.3%
10 h	Li_1.03_Co_0.99_PO_4_	10.216(3)	5.925(4)	4.706(1)	284.9	2.7	7.6%

**Figure 5:**
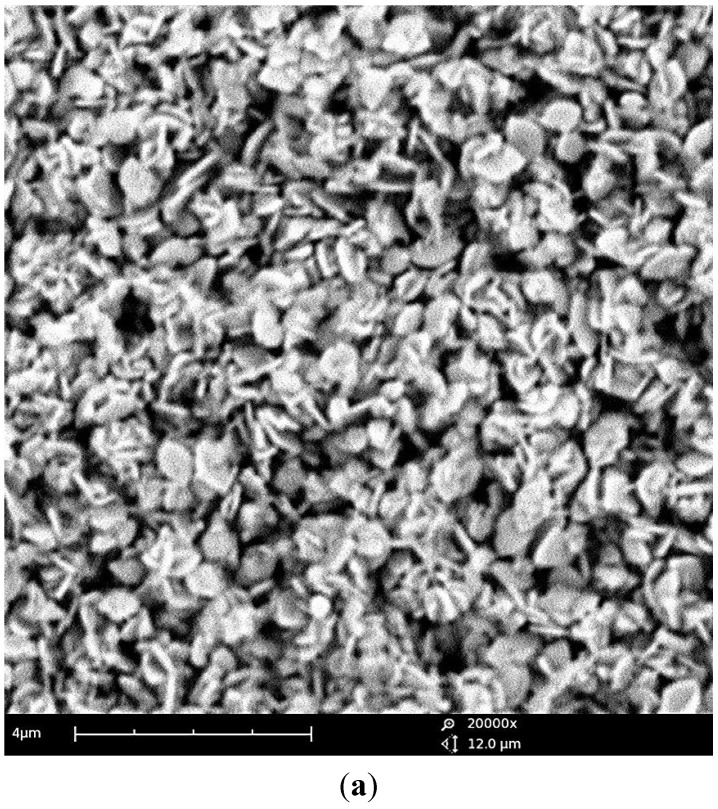

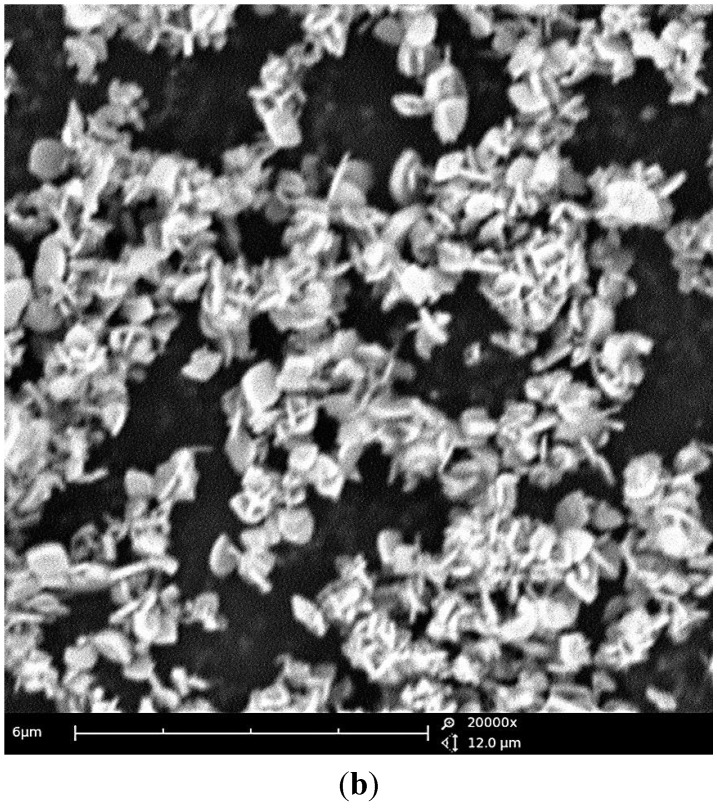
Scanning electron microscopy (SEM) micrographs of the LCP phase-pure samples obtained at 240 °C from the reagent ratio LiOH:LiH_2_PO_4_:CoSO_4_ = 2:1:1 after a reaction time of 5 h ((**a**) panel) and 10 h ((**b**)panel).

Turning to the morphological evolution upon reaction time, the particle size smoothly evolves after the precipitation of a phase-pure LCP material (4 h). The comparison between the particles’ morphology of the materials obtained at 240 °C after 5 h and 10 h is shown in the Figure 5.

A moderate increase in the size of the regular crystallites is observed with the increase of the reaction time. The LCP material obtained at 240 °C in 5 h is constituted by hexagonal sub-micrometric platelets with a thickness approximately below 200 nm, as estimated by the particle size routine using the ImageJ code [13], whereas after 10 h particles are slightly larger and thicker but without remarkable changes in the overall particles shape and morphological homogeneity.

**Figure 6:**
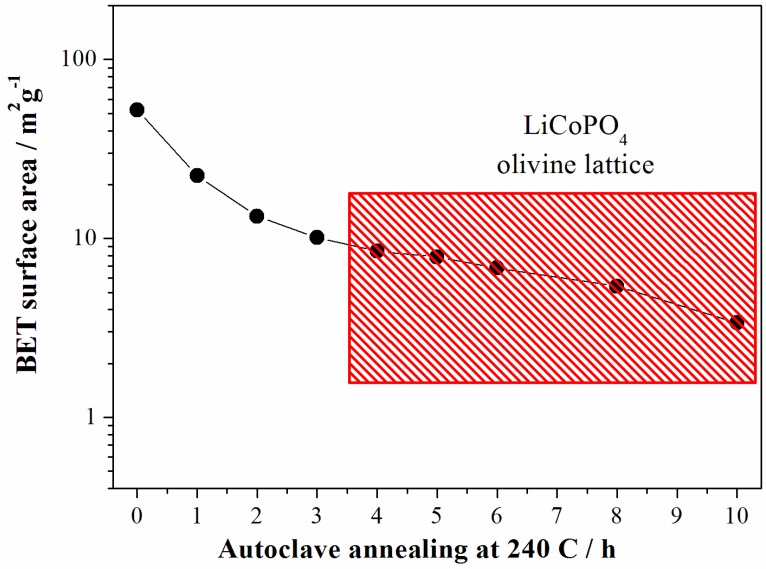
Evolution of the surface area of the LCP samples prepared at 240 °C from the reagent ratio LiOH:LiH_2_PO_4_:CoSO_4_ = 2:1:1. Data from samples crystallized in a LCP olivine lattice falls into the red rectangle.

As expected the increase of the particle size for longer reaction time results in the decrease of the sample surface area as shown in the Figure 6.

In summary, at 240 °C the LCP phase forms after 4 h of solvo-thermal treatment: the structures apparently do not evolve with longer reaction time, whereas the morphology of the particles continues to grow.

### 2.4. Analysis of the Synthesis Conditions: Effect of the Reaction Temperature

The effect of the temperature of the solvo-thermal bath has been tested by precipitating LCP-pure materials at three different temperatures, *i.e.*, 240, 220, and 200 °C, starting from the following reagent ratio: LiOH:LiH_2_PO_4_:CoSO_4_ = 1.75:1:1. Details of the synthesis conditions are reported in the Table 2. As expected, the adoption of different reaction temperatures implies different reaction kinetics. In this view, different solvo-thermal reaction time has been unavoidably optimized for each synthesis at different temperatures. The reaction time values reported in Table 2 are the minimal reaction times to obtain a phase pure LCP material for each temperature.

**Table 2:** Experimental conditions and structural refinements of samples prepared at different reaction temperatures in acidic pH conditions.

Reaction Temperature (°C)	Reaction Time (h)	Estimated Stoichiometry (from ICP Data)	a/Å	b/Å	c/Å	V/Å^3^ ± 0.3	%_Me Disorder_ ± 0.6	_w_*R*_p_
200	30	Li_1.01_Co_0.99_PO_4_	10.205(5)	5.923(4)	4.700(3)	284.1	1.6	8.5%
220	15	Li_1.01_Co_1.00_PO_4_	10.219(2)	5.926(2)	4.707(4)	284.9	2.9	2.1%
240	5	Li_0.99_Co_1.00_PO_4_	10.212(3)	5.922(3)	4.705(1)	284.5	2.5	7.7%

The XRD patterns of the three materials obtained at 240, 220, and 200 °C show phase-pure materials with olivine structure without contaminants: patterns are very similar to those shown in the Figure 3 (red lines) and have, therefore, been omitted to save space. The stoichiometries of the LCP phases for each sample derived from ICP-AES data are reported in the Table 2 together with the Rietveld Refinment results from the corresponding XRD pattern.

All preparations have line composition that matches, within errors, the theoretical LCP Li:Co = 1:1 stoichiometry. Materials prepared at 220 and 240 °C are very similar from the point of view of the cell volume and cationic disorder, whereas the sample prepared at 200 °C shows a slight lattice contraction, which occurs with the expected parallel decrease of the disorder [4].

**Figure 7:**
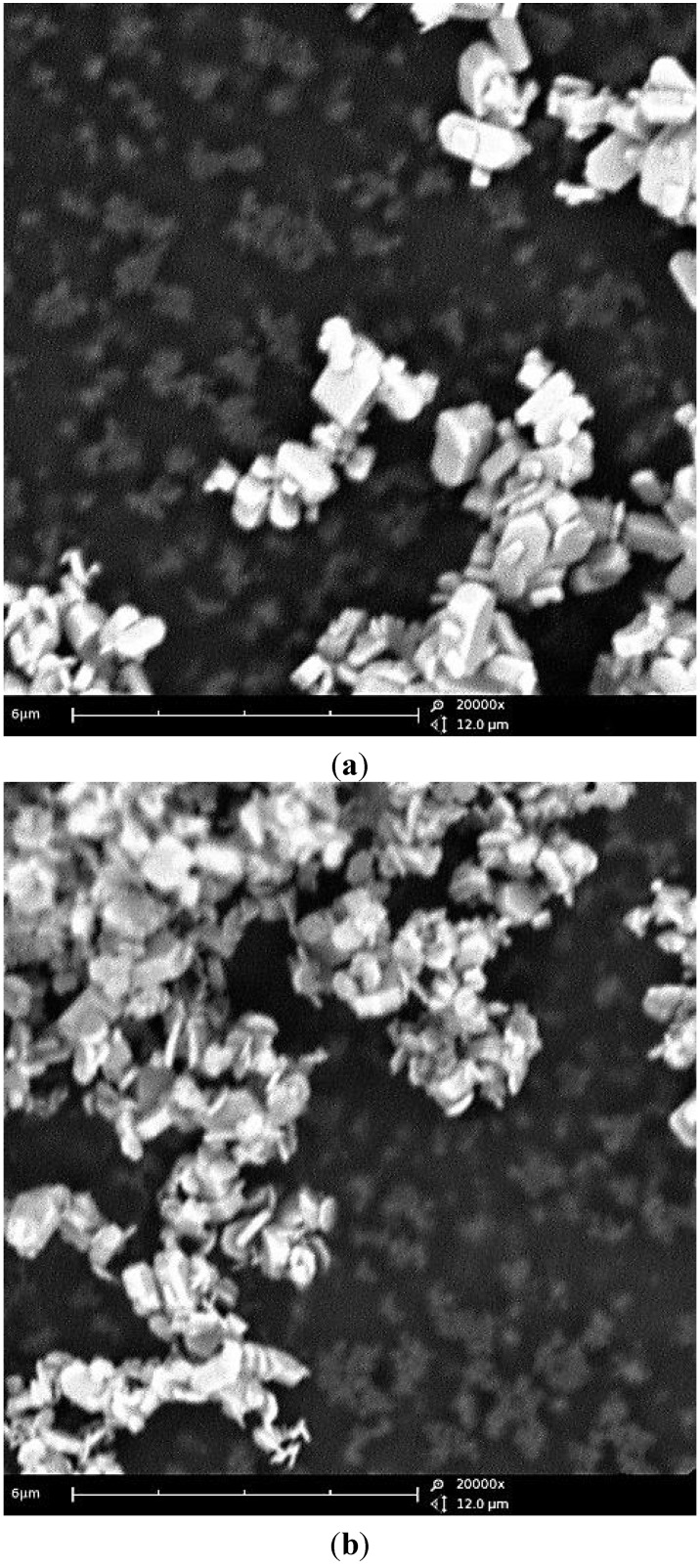

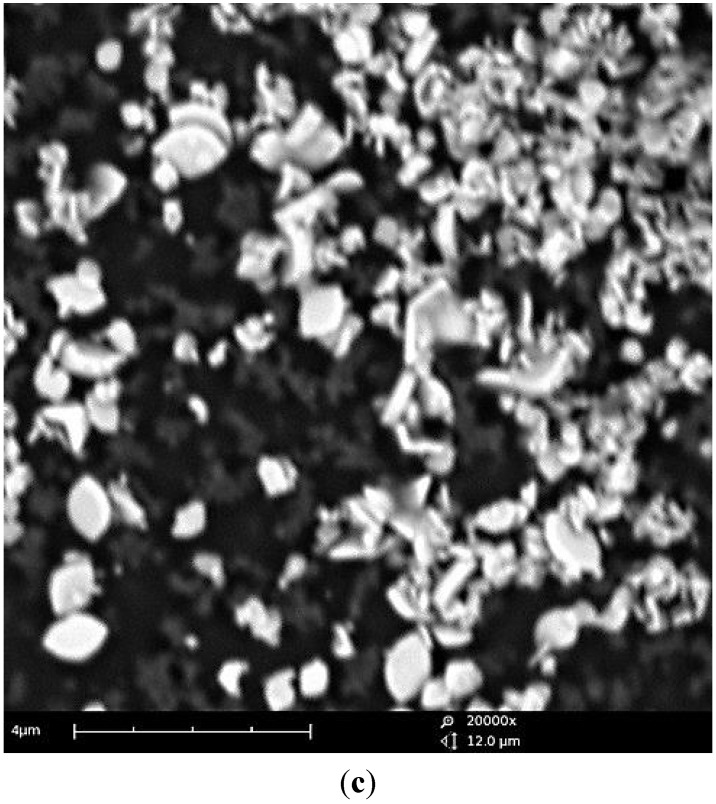
SEM micrographs of the materials prepared at (**a**) 200, (**b**) 220, and (**c**) 240 °C in acidic pH conditions.

The morphologies of the three materials are shown in the SEM micrographs reported in the Figure 7. Apparently the particle size and shape moderately depend on the solvo-thermal temperature. All materials are constituted by regular prismatic particles. Samples prepared at 240 °C (Figure 7c), are constituted by hexagonal-octagonal platelets, similar to those obtained at the same temperature but at neutral pH shown in the Figure 5. However, the morphological homogeneity and uniformity of the particle sizes are reduced compared to neutral pH samples.

On the other hand syntheses carried out at lower temperatures (220 and 200 °C) show slightly larger prismatic particles (especially thicker platelets) compared to the thin, regular octagonal/hexagonal platelets prepared at 240 °C. This trend is confirmed by the measured BET surface areas being 7.9 ± 0.2, 5.1 ± 0.1 and 3.8 ± 0.5 for the materials prepared at 240, 220, and 200 °C, respectively. One may speculate that syntheses carried out at lower temperature produce a smaller number of starting crystalline seeds due to a possible kinetic bottleneck in the nucleation activation energy. However, the study of the LCP crystal growth thermodynamics and kinetics is beyond the scope of this communication and therefore our hypothesis is presented only as mere speculation.

### 2.5. Analysis of the Synthesis Conditions: Effect of the Cobalt Anion

In their work about the hydrothermal growth of LFP olivine lattice Lu *et al.* [14] observed that the presence of charged species dissolved into the reaction bath directly affects the olivine crystal growth. In fact the different affinity of various anions and cations towards different lattice surfaces of the LFP particles leads to an effective selectivity to drive the final prismatic morphology. In order to shed light on this effect the LCP materials have been synthesized starting from several cobalt salts. Four different Co^2+^ sources have been used, *i.e.*, Co(NO_3_)_2_, CoSO_4_, Co(CH_3_COO)_2_, and CoCO_3_. The experimental conditions (reagent ratio, temperature, and reaction time) suitable to obtain the precipitation of pure LCP materials starting from the four different cobalt salts are summarized in Table 3. It is to be noted that shorter reaction times lead to the incomplete formation of the LCP phase with presence in the final material of phase impurities (cobalt phosphate, lithium phosphate). Moreover, it is important to underline the very long reaction time required to obtain an LCP-phase pure sample starting from cobalt carbonate. This extended reaction time (100 h) is uncommon in solvo-thermal and hydrothermal synthesis but, in this case, the low solubility of both reagents (cobalt carbonate) and products (LCP) apparently slow down the kinetics of the reaction strongly, compared to the other three cases where soluble cobalt salts are used as precursors.

All of the materials prepared in the conditions reported in the Table 3 are constituted by the pure LCP phase without contaminants: XRD patterns are very similar to those shown in the Figure 2 and Figure 3 (red lines) and have, therefore, been omitted to avoid redundancies. The results of the Rietveld refinements carried out on the XRD pattern of each sample are reported in the Table 4.

**Table 3:** Experimental conditions applied during solvo-thermal synthesis of the LCP samples starting from different cobalt salts (solvent volume 30 mL, reaction temperature 220 °C).

Co(II) Salt	Co Salt (10^−3^ mol)	LiH_2_PO_4_ (10^−3^ mol)	Sucrose (10^−3^ mol)	LiOH (10^−3^ mol)	Reaction Time (h)
Nitrate	3	3	0.1	5.25	15
Sulfate	3	3	0.1	5.25	15
Acetate	3	3	0.1	3	30
Carbonate	3	3	0.1	0.25	100

**Table 4:** Results of the structural refinements of samples prepared from different cobalt(II) salts.

Co(II) Salt	Reaction Time (h)	Estimated Stoichiometry (from ICP Data)	a/Å	b/Å	c/Å	V/Å^3^ ± 0.3	%_Me Disorder_ ± 0.3	_w_*R*_p_
Nitrate	15	Li_1.00_Co_0.99_PO_4_	10.205(2)	5.915(1)	4.700(1)	283.8	1.9	8.6%
Sulfate	15	Li_1.01_Co_1.00_PO_4_	10.219(2)	5.926(2)	4.707(4)	284.9	2.9	2.1%
Acetate	30	Li_1.00_Co_1.00_PO_4_	10.210(1)	5.922(1)	4.704(1)	284.4	1.7	6.1%
Carbonate	100	Li_1.00_Co_0.99_PO_4_	10.202(1)	5.915(1)	4.699(1)	283.6	1.7	7.7%

The stoichiometries of all samples have been estimated from ICP-AES data and correspond to an almost ideal Li:Co = 1:1 stoichiometry within errors. On the other hand the structures of three out of four olivine lattices are very similar: only the sample synthesized from the sulphate salt shows larger cell parameters, and the corresponding expansion of the cell volume, together with an increase of the metal disorder between Li and Co sites.

**Figure 8:**
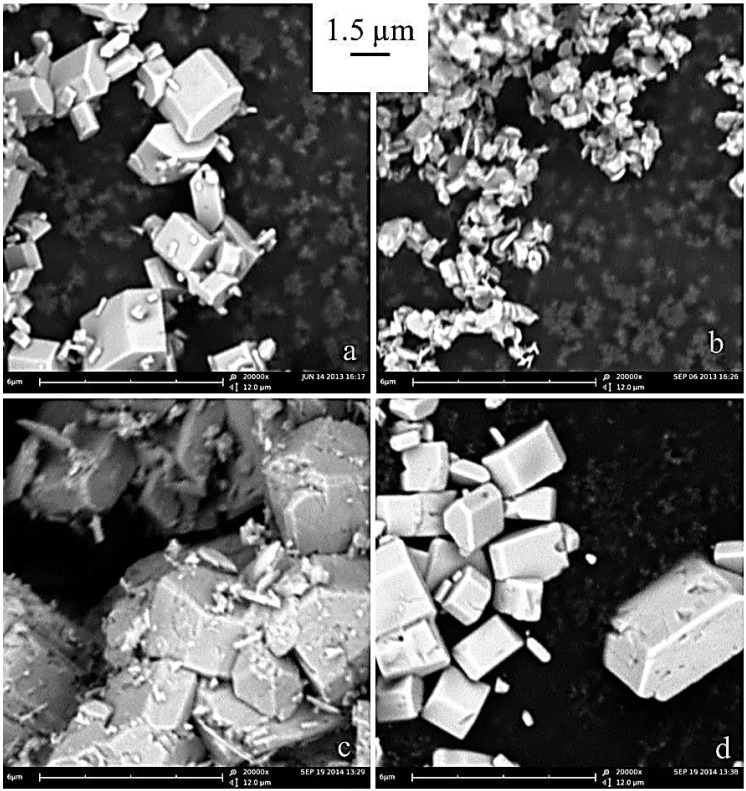
SEM micrographs of the prepared LiCoPO_4_ powders: (**a**) nitrate; (**b**) sulfate; (**c**) acetate; and (**d**) carbonate.

Similarly, the morphologies of the four samples can be grouped, putting together materials prepared from nitrate/acetate/carbonate precursors opposite to the LCP material synthesized from CoSO_4_. The SEM micrographs of the four samples are shown in the Figure 8.

As already discussed the LCP sample prepared from cobalt sulfate is constituted by octagonal/hexagonal thin platelets. On the contrary the three materials prepared from nitrate/acetate/carbonate precursors show large isotropic prismatic crystallites with sizes above 1–2 mm in all the crystallographic directions. In particular, samples prepared from cobalt nitrate and carbonate are constituted by regular and well-formed prisms with hexagonal bases.

The LCP material prepared from cobalt acetate is also constituted by very large isotropic prisms, but with less clear prismatic regularities and with a significant morphological impurities (*i.e.*, small irregular particles spread on the surfaces of the prisms).

These results are in very nice agreement with the findings of Lu *et al.* [14] for the growth of LFP crystals in hydrothermal conditions. As already discussed, these authors observed that different anions (sulfate and nitrate, in their case) have different absorption ability on different lattice planes of the same olivine crystals. This selective absorption apparently drives the crystal growth along specific crystal directions as an example by inhibiting or promoting the precipitation on the (101) plane [14]. Additionally, in the case of the LCP preparation in solvo-thermal conditions, the use of sulfate salts apparently inhibit the crystal growth perpendicular to the (101) crystal facet, thus leading to the precipitation of octagonal/hexagonal platelets very similar to those observed by Lu *et al.* for LFP crystals from sulfate precursors [14].

In summary, our evidence confirms that the tuning of a crystal growth in a specific lattice direction or its limitation as well as the number of nucleation sites are strongly altered by the nature of the cobalt counter-anion in the precursor salt. As already mentioned, a similar effect has been reported by Lu *et al.* [14] for LFP materials and here we also confirm this trend for the LCP crystal growth in solvo-thermal conditions.

On the contrary, a simple effect of the nature of the cobalt salt precursors on the lattice ordering in the final LCP phase is not straightforward. In fact, although the choice of specific anions seem to drive crystallization with improved cation ordering, since the experimental synthesis conditions are necessarily different for different salts, it is not possible to draw a simple cause-effect rule.

### 2.6. Electrochemical Tests of the LCP-Pure Samples in Li Cells

In the previous sections a number of different preparation recipes have been reported to obtain phase-pure LCP materials with slightly different morphological identity and homogeneity, structural properties, and composition. A summary of the synthesized materials is presented in the Table 5.

**Table 5:** LCP phase-pure materials prepared with different solvo-thermal recipes.

Sample	LiOH/Co^2+^ Salt Ratio	Reaction Time (h)	Reaction Temperature (°C)	Co(II) Precursor
LCP-01	2:1	5	240	Sulfate
LCP-02	1.75:1	5	240	Sulfate
LCP-03	2:1	4	240	Sulfate
LCP-04	2:1	6	240	Sulfate
LCP-05	2:1	8	240	Sulfate
LCP-06	2:1	10	240	Sulfate
LCP-07	1.75:1	15	220	Sulfate
LCP-08	1.75:1	30	200	Sulfate
LCP-09	1.75:1	15	220	Nitrate
LCP-10	1:1	30	220	Acetate
LCP-11	0.083:1	100	220	Carbonate

In this section the electrochemical performances of the 11 synthesized materials are compared in order to highlight the effect of the various preparation conditions on the ability to reversibly exchange lithium in a Li-cell.

**Figure 9:**
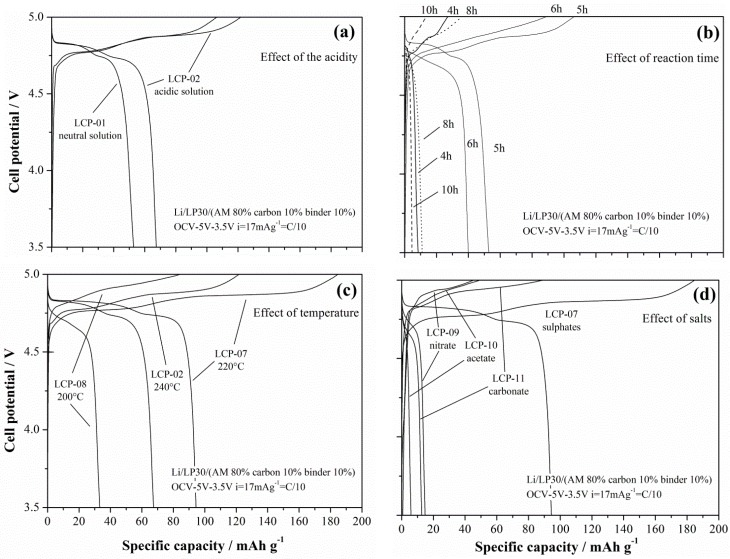
Lithium de-insertion/insertion curves from galvanostatic cycling of the LCP materials.

The lithium de-insertion/insertion curves for galvanostatic cycling at 17 mA·g^−1^ (C/10) are shown in Figure 9a–d.

The LCP material reversibly cycles lithium ions at approximately 4.8 V *vs.* Li: the oxidation/reduction steps show two plateaus in agreement with the reaction mechanism by Bramnik [15]. Apparently, different synthesis conditions lead to different performances in lithium cells. In particular, acidic solvo-thermal solutions improves the performance, as well as assessed reaction times. Concerning this last effect, the occurrence of an optimal reaction time to achieve the best performances has also been observed by Lu *et al.* [14] for similar synthesis of the LFP olivine. Lu *et al.* put this behavior in direct correlation with two competing effects, *i.e.*, the ordering of the olivine lattice and the crystal growth that improves and decreases the ability of reversibly-exchanged lithium, respectively. Furthermore, for the here-synthesized LCP materials, both of these parameters are apparently playing a similar role: in fact, the cationic disorder is estimated to reduce slightly after 5 h of synthesis without further signficant changes for longer reaction times, whereas the size of the prismatic particles increase, thus reducing the surface area of the material. These findings are also in agreement with the observation reported by Truong and coworkers in References [16].

Additionally, the trend of the performances in lithium cells *vs.* the synthesis temperature show an interesting optimum value. In fact, the LCP-07 material that has been synthesized at 220 °C shows improved performance compared to both the materials prepared at higher (LCP-02 at 240 °C) and lower (LCP-08 at 200°C) temperatures. In this case a clear explanation of this trend is missing, since apparently the competition between the lattice ordering and the crystallite size cannot account for this peculiar behavior. One may speculate that, at different temperatures, the growth of the crystal facets with different *hkl* Miller index may be different, as well as the number of nucleation sites of the prismatic particles. Therefore, as a mere hypothesis, one may suggest an optimal reaction temperature may result from the balancing between the nucleation thermodynamics and crystal growth kinetics.

Turning to the effect of the cobalt salt precursor, apparently the selection of different anions has a massive impact on the ability of the resulting LCP materials to reversibly cycle lithium in cells. On the other hand, this behavior is expected due to the very large difference in morphology between the four compared materials. In fact the only LCP material with crystallite size below 1 micron is the only electrochemically active one, whereas all micro-sized samples show negligible performances, likely due to the much-extended diffusion paths within the LCP lattice for the lithium ions to be extracted or inserted in the structure. Lithium diffusion is a well-known limiting factor for olivine-based materials [4,5] in particular for LCP phases, thus the need of sub-micro-sized particles to achieve acceptable electrochemical performances.

**Figure 10:**
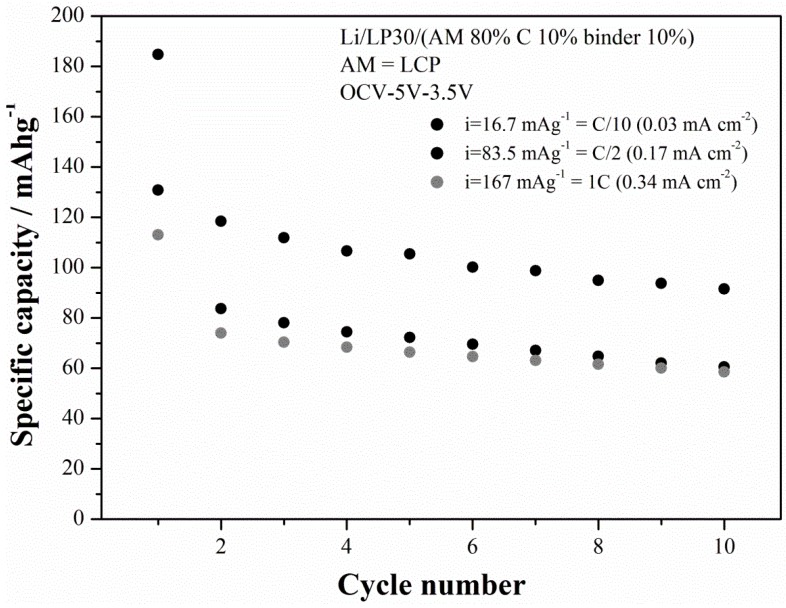
Cycling performance (charge capacities) of the LCP-07 material prepared at 220 °C under moderately-acidic conditions for 15 h.

Among all the synthesized LCP materials the outperforming one is LCP-07 that has been prepared starting from cobalt sulfate in a moderately acidic solution treated at 220 °C for 15 h. The cycling performance at three different current rates is shown in the Figure 10 for the LCP-07 material.

The capacity in the first charge at C/10 slightly exceeds the theoretical capacity (167 mAh·g^−1^), thus suggesting the occurrence of the electrolyte decomposition at high voltage. In fact EC:DMC electrolytes with fluorinated salts are at the limits of their stability window and have been reported to decompose on LCP-based electrolyte even with the use of stabilization additives [5,17]. This hypothesis is also confirmed by the value of the coulombic efficiencies in the first cycle, *i.e.*, 51%, as well as by the fading trend of the specific capacities upon cycling. The last effect has been recently put in correlation with the occurrence on an apparently unavoidable increase of the cationic disorder within the olivine lattice [18]. On the other hand, it is to be noted that, at C/10, the ability of the material to supply a specific capacity upon cycling approaches 90–100 mAh·g^−1^ after 10 cycles. At higher current rates performance drops to approximately 1/3 of the theoretical performance. However the deterioration of the capacity performance at high rates is likely due to the poor electronic conductivity of this material [5] and may be mitigated by the growth of carbon coatings or doping, similarly to the parent LFP-phase [4,19]. In this view this solvo-thermal synthetic route allows the preparation of a material with large room for improvements.

## 3. Experimental Section

Samples have been characterized by a multi-technique approach in order to investigate the materials elemental compositions, the crystal structure by X-ray diffraction, the sample morphology by scanning electron microscopy and N_2_ absorption, the materials’ vibrational properties by Raman and infrared spectroscopy, and the electrochemical performances in lithium half cells.

The composition of the LCP materials has been measured by inductively-coupled plasma-atomic emission spectroscopy (ICP-AES) by means of a Vista MPX Rad-VARIAN instrument (Palo Alto, CA, USA). The samples were digested by addition of nitric and sulfuric acid (HNO_3_, 70% and H_2_SO_4_, 96%) and, finally, dissolved in deionized water to obtain concentration of Li and Co in the order of 10^−4^ M.

Sample morphology has been investigated by scanning electron microscopy (SEM) using a Phenom-FEI instrument (Hillsboro, OR, USA). Fast-Fourier Transform infrared spectroscopy (FTIR) experiments have been carried out by the Bruker Alpha Instrument (Ettlingen, Germany) in transmission mode on CsI pellets.

XRD experiments have been carried out by using a X’Pert pro MPD diffractometer (Panalytical, the Netherland) and a Cu Kα radiation source. XRD data have been collected with steps of 0.025°, with a time step of 8.2 s on a powder sample spread on a glass sample holder. Structure refinements have been performed through the Rietveld method by using GSAS code starting from the olivine lattice of LiCoPO_4_ [9]. Refinements have been carried out starting from the olivine prototypal lattice [5] (orthorhombic unit cell with space group n°62 Pnma, Li atoms in 4a (0, 0, 0), Co in 4c (x_TM_, ¼, z_TM_), P in 4c (x_P_, ¼, z_P_), O in 4c (x_O_, ¼, z_O_) 4c′ (x_O′_, ¼, z_O′_), and 8d (x_O″_, y_O″_, z_O″_)). Considering the low scattering factors of lithium and oxygen, few constraints have been adopted in order to carry out reliable structural refinements. In particular, the stoichiometries of the various samples have been fixed (occupancies have not been optimized), and the Debye–Waller factors have been assumed equal for all cations (Li, Co, Fe) and for the PO_4_^3−^ atoms (P, O), and refined simultaneously for all atoms. In summary, in the Rietveld refinement procedure the following parameters have been optimized: (a, b, c) lattice parameters, Caglioti peak-broadening coefficients, and Gaussian-to-Laurenzian peak shape coefficients, the atomic positions, the cations and the PO_4_^3−^ thermal factors, and the anti-site Li/Co cationic disorder. Sample stoichiometries have been estimated starting from the ICP-AES data by assuming a Li^+^/Co^2+^/PO_4_^3−^ electro-neutrality constraints.

Surface area measurements have been carried out by single point N_2_ absorption using a Monosorb Quantachrome instrument. All samples have been outgassed under N_2_/He flow for 12 h at 120 °C before measurements.

Electrode casting has been deposited on an Al foil by doctor blading a slurry composed of 80% of the active material (AM), 10% of PVdF-HFP (Kynar Flex 2801), and 10% of Super P carbon in tetrahydrofuran (THF, Sigma-Aldrich, St Louis, MO, USA). The mass loading over the aluminum foil is approximately 2–3 mg·cm^−2^. All the samples have been tested by galvanostatic cycling in lithium cells in the cell potential range of 3.5–5 V at 0.1C rate (1C = 167 mA·g^−1^). The galvanostatic cycling experiments have been carried out with a MTI galvanostat using Swagelok-type cells. The cells have been prepared in an Iteco Engineering Ar-filled glovebox, by coupling the electrode under test with a lithium foil counter-electrode in 1 M LiPF_6_ ethylene carbonate/dimethylcarbonate (EC:DMC) electrolyte solution (Solvionic), soaked on a Whatman™ glass fiber separator.

## 4. Conclusions

The analysis of the effect of the synthesis conditions on the preparation of LCP phases from solvo-thermal solutions has been carried out by a multiple-technique approach. XRD, SEM, BET, and FTIR characterizations of the materials have been carried out in order to draw a comprehensive picture of the physical-chemistry properties of the synthesized materials. Four different reaction parameters have been evaluated: acidity of the solvo-thermal solution, reaction time, reaction temperature, and effect of the chemical nature of the cobalt precursor salt. All of these parameters drive peculiar aspects of the solvo-thermal precipitation of the LCP phases. In particular, the acidity of the solution affects the morphological homogeneity; longer reaction time slightly enlarges the crystal size; lower reaction temperatures lead to fewer and larger crystallites; and different cobalt counter-anion in the precursor salts greatly enhance/limit the crystal growth. This last parameter has, in fact, the most evident effect in the promotion/limitation of the precipitation of well-formed and large LCP prismatic crystals.

On the other hand, in lithium cells, the outperforming samples are, as expected, those with sub-micrometric crystallites with platelet shape orthogonal to the (010) crystal orientation. This morphology results in a remarkable electrochemical activity of the prepared materials; in particular, those grown under acidic conditions at 220 °C for 15 h.
